# Self-template/activation nitrogen-doped porous carbon materials derived from lignosulfonate-based ionic liquids for high performance supercapacitors†

**DOI:** 10.1039/d0ra06821g

**Published:** 2020-10-05

**Authors:** Qinqin Xu, Xia Wang, Jian Cheng, Lin Zhang, Feng He, Haibo Xie

**Affiliations:** Department of New Energy Materials & Engineering, College of Materials & Metallurgy, Guizhou University Huaxi District Guiyang 550025 P. R. China hbxie@gzu.edu.cn

## Abstract

A simple ion exchange reaction of sodium lignosulfonate (SLS) and 1-allyl-3-methyl imidazolium chloride ([Amim]Cl) produced a new polymeric ionic liquid [Amim]LS and NaCl, and the mixture was successfully used as a precursor to prepare a nitrogen-doped porous carbon material *via* direct carbonization without any additional activation agent or template. It was believed that the *in situ* produced NaCl during the precursor synthesis process acted as the self-template and in self-activation. The introduction of imidazolium ionic liquid into the precursor raised the nitrogen content of the obtained carbon material up to 4.68% for a high yield of [Amim]LS-700 carbon material up to 34.6%. The effect of carbonization temperature on the structures and electrochemical properties of the prepared carbon were also studied systematically. It was found that the carbon material exhibits a superior gravimetric capacitance up to 230 F g^−1^ (0.1 A g^−1^) at the carbonization temperature of 700 °C, a good energy density of 7.99 W h kg^−1^ at the power density of 25 W Kg^−1^, and an excellent cycling stability of 90.3% after 20 000 cycles. This work provides a new path for the value-added utilization of biomass coupled with the field of electrochemical energy storage.

## Abstract 2

Without any additional template or activation agent, a high N-doped porous carbon was easily prepared by a simple ion exchange reaction and a following carbonization, and showed excellent electrochemical performance as a supercapacitor electrode.

## Graphical abstract

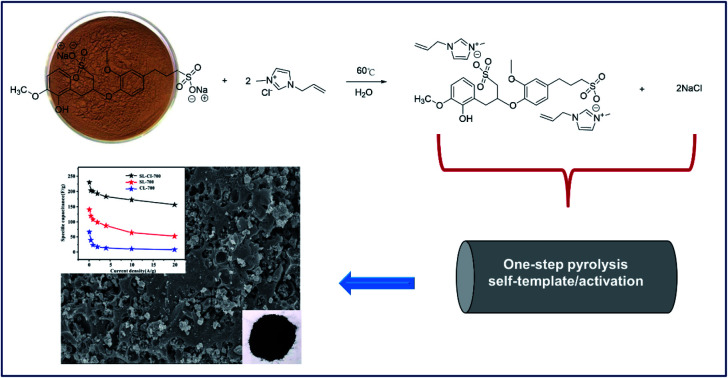

## Introduction

Supercapacitors are well known as novel energy storage systems between secondary batteries and physical capacitors, and have the characteristics of high energy density, wide operating temperature range, fast charge/discharge with high coulomb efficiency and long cycle lifetime.^1,2^ Supercapacitors are categorized into double-layer capacitors (EDLCs) and pseudocapacitors on account of energy storage. The energy storage of EDLCs is due to the facile and reversible adsorption–desorption of electrolyte ions on the electrode surface to form an electrical double layer, and the capacitance storage of a pseudocapacitor is through the redox reaction that occurs on the surface of the electrode material.^3,4^

In the past few years, carbon materials as electrode materials have received extensive attention to develop energy storage of EDLCs on account of their low cost, good conductivity, excellent chemical stability and long cycle life.^5,6^ However, low capacitance and energy density of EDLCs limits their application on high performance requirements for industrialization. Currently, the energy density of activated carbon for commercial electrode materials of supercapacitors is mainly in the range of 4 to 5 W h kg^−1^, much lower than that of lead acid batteries being of about 26 to 34 W h kg^−1^.^7,8^ Therefore, many studies on modified carbon materials have been carried out to improve the energy density of EDLCs, such as carbon fibre,^9,10^ templated carbon,^11,12^ graphene,^7,13^ carbon nanotubes^14–16^ and porous carbon,^6,17^*etc.* Porous carbon materials, with high specific surface area, abundant channel structure, adjustable aperture and good conductivity, have received much attention to be electrode materials of supercapacitors.^17,18^ It is well known that the performance of EDLCs can be improved with (i) large specific surface area coupled with reasonable pore structures; this can promote ions transfer through electrode surface and electrolyte and (ii) doping of heteroatoms or introducing special functional groups to the carbon materials; this can enhance the hydrophilicity of carbon materials and produce additional pseudo capacitance through redox reaction by increasing reactivity.^19–22^ Therefore, the development of new methods of improving specific surface area, optimizing pore size distribution, and introducing heteroatoms into carbon materials are desirable to improve electrochemical performance of supercapacitors.

Generally, in order to obtain large specific surface area and form hierarchical pore structures, most of the carbon materials need to use hard/soft templates such as zeolites,^11,12^ silica template^23^ and to be chemically activated by KOH or NaOH,^24,25^ ZnCl_2_,^26–28^ K_2_CO_3_ (ref. 29 and 30) and Na_2_CO_3_,^31^*etc.* However, these activation agents suffer from issues such as high cost, corrosion and low materials yield, which limit their further use in industrial application.^32^ Recently, more attention has been paid to new, facile and sustainable activation preparation methods to prepare porous carbon materials. Yang *et al.* prepared N, O and S co-doped hierarchical porous carbons by directly pyrolyzing alkaline lignin without any additional activation agents, and the impurities in alkaline lignin such as NaCl, KCl, Na_2_CO_3_ and Na_2_SO_4_ were found to play an important role in the formation of hierarchical porous carbons.^33^ Wang *et al.* reported a N-doped porous carbon by one-step self-activation method which benefited from the celery's internal water transport system.^34^ With specific surface area of 1186 m^2^ g^−1^ and the average diameter of 0.94 nm, the capacitance of the carbon material was 245 F g^−1^ at 0.2 A g^−1^. Zheng *et al.* developed a self-activation method to prepare porous carbon from silkworm cocoon and phytic acid.^35^ It was found that the decomposition of phytic acid during carbonization was beneficial to the formation of pore structure. Mesopores with 50–100 nm were caused by the self-assembly of silkworm, which facilitated ion transport. Xu *et al.* prepared porous carbons by pyrolyzing the ethylene diaminetetraacetic acid disodium zinc salt directly.^36^ The nano-ZnO and Na_2_CO_3_ from precursors were found to act as self-template for the carbon materials. The specific surface area of the prepared carbon materials reached at 1368 m^2^ g^−1^ and the capacitance of the carbon sample was 275 F g^−1^ at 0.1 A g^−1^ (in 6 M KOH). Therefore, self-templating and self-activation has shown more prospects as a green and sustainable strategy to prepare porous carbon materials for supercapacitor electrode materials.

Derived from a large amount of waste produced in the pulping process, sodium lignosulfonate has many advantages such as low price, high carbon content, good thermal stability. Sodium lignosulfonate contains various functional groups such as phenolic groups, carboxyl groups and sulphur groups,^37,38^ but without nitrogen element. It was reported that N-containing porous carbon materials could be prepared by directly pyrolyzing sodium lignosulfonate without additional templating and activation, in which NaCl and Na_2_SO_4_ were considered to act as the self-template and self-activation roles;^26^ however, the content of doped nitrogen was very low, which came from the impurities of the sodium lignosulfonate source. It is well known that the nitrogen/nitrogen-containing functional groups can not only increase the ion transfer rate, but also facilitate the internal redox reaction which improves the pseudo-capacitance of electrode materials.^39^ It is still desirable to seek for real effective and sustainable methods to prepare N-doped porous carbon derived from sodium lignosulfonate.

Being as a special class of molten salts formed by the extensive combination of cation and anion, ionic liquids were considered to be excellent precursors for preparation of N-doped carbon materials due to their outstanding properties such as low volatility, super electrical conductivity and thermal stability, and especially convenient molecular structure tunability,^40–42^ which contribute to the preparation of carbon materials with high N content and good electrical conductivity.^40,43,44^ From the perspective of molecular design and taking the merits of both of using biomass and relatively cheap protic ionic liquids as precursors, we recently reported a facile and structure tunable preparation method of precursors for preparation of N-doped porous carbon materials, which exhibited ultrahigh surface area, high N-doping content and showed good supercapacitor performance.^45–47^

It is well know that the preparation ionic liquids with various anions usually use halogen-based anion ionic liquids as precursors through anion exchange reaction with corresponding organic or inorganic salts, in association with the yielding of halogen inorganic salts. For example, the preparation of DBU-based ionic liquids with benzenesulfonate anions.^48^ We conjectured that if we used the as-prepared ionic liquids without separating the by-produced inorganic salts as a percusor for carbon materials, the inorganic salts could be act as a self-template and self-activation agent during the porous carbon materials preparation.^36,49,50^

**Scheme 1:**
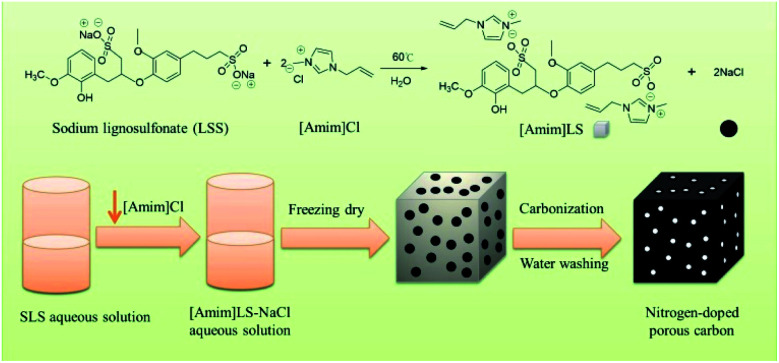
The synthesis of [Amim]LS precursors for N-doped porous carbon materials preparation.

In this study, taking the advantage of SLS molecular structures, we prepared a new polymeric ionic liquid [Amim]LS through a simple ion exchange reaction between sodium lignosulfonate (SLS) and 1-allyl-3-methyl imidazolium chloride ([Amim]Cl), in which NaCl was produced as a by-product. And then the produced [Amim]LS–NaCl mixture was used successfully as a precursor to obtain N-doped porous carbon materials *via* direct carbonization without any additional activation agent or template (Scheme 1). The as-prepared carbon materials had higher N and O content and much more excellent electrochemical performance than the carbon materials derived from pure lignosulfonate. The effect of carbonization temperature on the carbon structures and electrochemical properties were also studied systematically. It was found that the carbon material exhibits excellent gravimetric capacitance of 230 F g^−1^ (at 0.1 A g^−1^) at the carbonization temperature of 700 °C, good energy density of 7.99 W h kg^−1^ at a power density of 25 W kg^−1^, as well as outstanding cycling stability of 90.3% after 20 000 cycles. The work provides a new path for the value-added utilization of biomass coupled with the field of electrochemical energy storage, taking advantages of ILs preparation as well as the use of biomass and ILs as precursors for porous carbon materials preparation.

## Experimental

### Materials

Sodium lignosulfonate (SLS) was purchased from Aladdin. 1-Allyl-3-methyl imidazolium chloride ([Amim]Cl) was obtained from Lanzhou Institute of Chemical Physics, Chinese Academy of Sciences. Potassium hydroxide (KOH, wt> 85%) was purchased from Sinopharm Chemical Reagent Co., Ltd.

### Preparation of SL and CL precursor

SLS (16.85 g) was dispersed in 80 mL deionized water firstly. Then [Amim]Cl (10.00 g) was added and stirred for 12 h at 50 °C. Finally, the solvent was removed by freeze-drying at −58 °C for 48 h, giving a dark brown solid.

### Preparation of porous carbon materials with [Amim]LS

The as-prepared carbon materials were obtained by one-step carbonization. The sample was heated to 600–900 °C at 5 °C min^−1^ and held for 2 hours. Then the samples were cooled to room temperature. All processes were carried out in an Ar atmosphere (200 mL min^−1^). The carbon materials were washed with 2 mol L^−1^ hydrochloric acid solution and then washed with deionized water several times to remove the inorganic salts. Afterwards, the sample was dried in vacuum at 60 °C for 12 h. The as-prepared carbon materials were named as [Amim]LS-*X*, where *X* represents different carbonization temperature from 600–900 °C.

For a comparative purpose, the carbon materials from single sodium lignosulfonate and 1-allyl-3-methyl imidazolium chloride were also prepared by carbonization with the same preparation condition as [Amim]LS-700, which were named as SLS-700 and [Amim]Cl-700 respectively, used for the comparison research.

### Materials characterization

The surface morphology of carbon materials was obtained under Hitachi Regulus 8100 scanning electron microscope (SEM). The Fourier transform infrared spectrometer (FTIR) was operated on Nicolet iS50. X-ray diffraction (XRD) was investigated using an X′ Pert PRO with Cu Kα_1_ radiation. Raman spectra were collected on Renishaw Invia with excitation wavelength of 532 nm to identify degree of graphitization. Surface element analysis of materials was carried out by X-ray photoelectron spectroscopy on Thermo Fisher K-Alpha with Al K Alpha. BET specific surface area (SSA) and porosity of the material were carried on Micromeritics ASAP 2460. The pore size distribution (PSD) was calculated on NLDFT Model by the N_2_ adsorption isotherms and the SSA was obtained by the Brunauer–Emmett–Teller (BET).

### Electrochemical measurements

The electrochemical properties of [Amim]LS-*X* were studied on a CS2350 electrochemical workstation (Wuhan Corrtest Devices Corp., Ltd, China) utilizing a standard two-electrode system in 6 M KOH aqueous electrolyte. The electrodes were fabricated by mixing a mass ratio of 85 : 10 : 5 ([Amim]LS-X : carbon black : PTFE) and pressed onto a nickel foam (1.1 cm^2^) for 5 s at 7 MPa. Then the electrode was vacuum-dried for 10 h at 100 °C. The mass loading of active materials on nickel foam was 4.05–4.20 mg cm^−2^. The two electrodes were separated by a NKKMPF30AC-100 cellulose membrane infiltrated with 6 M KOH aqueous electrolyte, and then a CS2032 button cell was used as a supercapacitor model. Cyclic Voltammetry (CV) and electrochemical impedance spectroscopy (EIS was from 0.01 Hz to 100 kHz with a voltage amplitude of 5 mV) measurements were performed on CS2350 electrochemical work-station (Wuhan Corrtest Devices Corp., Ltd, China) with potential ranging from 0 to 1.0 V in aqueous electrolyte. Galvanostatic Charge–Discharge (GCD) test was performed on LANHECT2001A.

## Results and discussion

### Synthesis and characterization of [Amim]LS precursors

The [Amim]LS precursor was synthesized by ion exchange reaction between SLS and [Amim]Cl with the procedure described in Scheme 1. Fig. S1† shows the FTIR spectra of both SLS and the synthesized polyionic liquid precursor ([Amim]LS). It was found that [Amim]LS is mainly caused by the external bending vibration of various C–H bonds in the range of 700–1000 cm^−1^. The peaks of the in-plane bending vibration in the C–H plane on the imidazole ring is at 940 cm^−1^, and the peaks at 1595 and 1451 cm^−1^ are typical aromatic ring C=C bonds in lignin sulfonates. The peaks at 1168 and 1100 cm^−1^ are assigned to the stretching vibration bands of [Amim]LS, indicating the successful preparation of [Amim]LS.

**Fig. 1:**
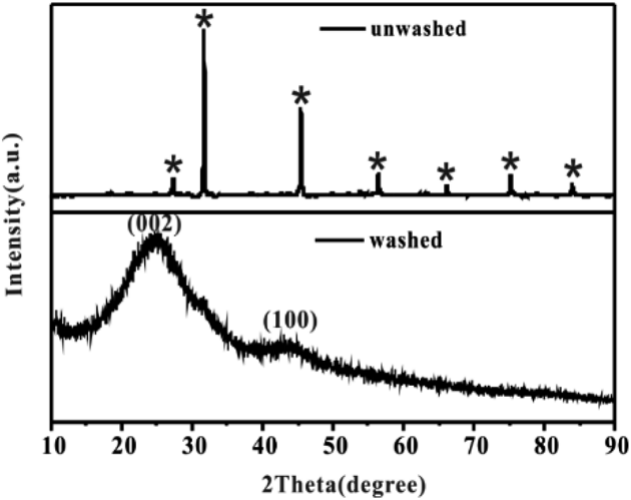
XRD patterns of [Amim]LS-700 carbon products before and after washing, where* stands for the typical diffraction peaks of NaCl (JCPDS card no. 05-0628).

After the successful synthesis of [Amim]LS–NaCl mixture precursors, a series of [Amim]LS-*X* carbon materials were prepared by carbonization of the synthesis products as shown in Scheme 1 directly at different temperatures. Fig. 1 shows the XRD patterns of [Amim]LS-700 carbon products before and after washing with HCl aqueous solution and deionized water. The diffraction peaks of NaCl were clearly found in the unwashed carbon material, while the typical diffraction peaks of NaCl disappeared completely after washing, indicating the presence of NaCl. It was found that inert chloride salts such as NaCl and KCl can be used as self-templates and/or self-activation agents for the porous carbon materials in previous publications.^49–51^ Therefore, it was considered that the keeping of NaCl in the precursors for carbonization is beneficial for the development of porous structures of the carbon materials, which will also be explicated in our discussion. However, it needs to point out that all the [Amim]LS-*X* carbon materials in the following discussion were obtained after the removal of NaCl in the samples by washing, in order to avoid any possible disturbance to the materials properties that may be caused by NaCl.

**Fig. 2:**
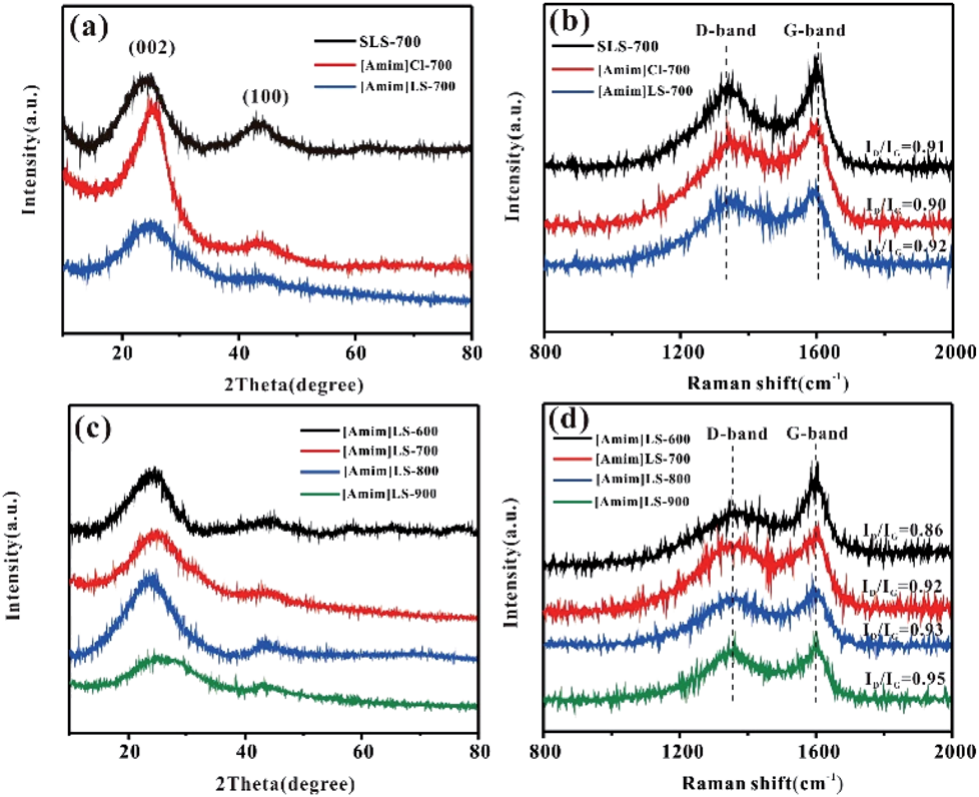
(a) XRD patterns and (b) Raman spectra of the SLS-700, [Amim]Cl-700 and [Amim]LS-700; (c) XRD patterns and (d) Raman spectra of the [Amim]LS-*X*.

The physical structures and properties of the carbon materials were investigated by XRD and Raman spectroscopy. As the XRD results shown in Fig. 2(a), there are two diffraction peaks clearly observed at about 24° and 43° on all the samples of [Amim]LS-700, SLS-700 and [Amim]Cl-700, representing the characteristic (002) and (100) crystalline faces respectively. The sharp characteristic shoulder peak at (002) in [Amim]Cl-700 indicates the good crystallinity of the sample. As contrast, the two broad peaks at 24° and 43° of [Amim]LS-700 are much weaker than those of [Amim]Cl-700 and SLS-700, suggesting that graphitized structure is only partially formed with the addition of [Amim]Cl to sodium lignosulfonate. Both D-band (1350 cm^−1^) and G-band (1580 cm^−1^) are observed in the Raman spectra of SLS-700, [Amim]Cl-700 and [Amim]LS-700 (Fig. 2(b)), of which the former band is attributed to the disorder induced by out-of-plane vibration structural of carbon skeleton defects, and the latter band is assigned to the plane vibration of sp^2^ in the graphitized C atom of the material. The relative intensity ratio (*I*_D_/*I*_G_) of the D-band and G-band is proportional to the degree of disorder of carbon materials. The *I*_D_/*I*_G_ values of SLS-700, [Amim]Cl-700 and [Amim]LS-700 is calculated to 0.91, 0.90 and 0.92, respectively, implying that the introduction of IL to sodium lignosulfonate increases the disorder degree by producing porous structure, but also maintain the graphite structure by reorganizing carbon atoms. Fig. 2(c) and (d) show the XRD and Raman analysis on the [Amim]LS-*X* carbon materials. In Fig. 2(c), two broad amorphous diffraction peaks at ∼23°and ∼43° are observed on all the [Amim]LS-*X* carbon materials, implying their amorphous carbon structure. As the Raman spectra shown in Fig. 2(d), [Amim]LS-600 present the smallest *I*_D_/*I*_G_ value of 0.86, indicating a high degree of graphitization, which could be owing to the low activation behavior of NaCl at relative low temperature of 600 °C. With the increase of pyrolysis temperature, the degree of graphitization of the material decreased slightly, which might be due to the reason that the inorganic salts in the precursors can produce and activate more pores with the temperature increasing, which leads to the disorder degree enhanced in the samples.

**Fig. 3:**
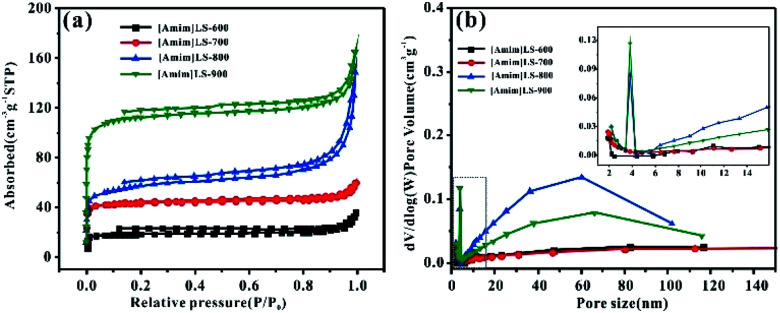
(a) N_2_ adsorption–desorption isotherm and (b) pore size distribution of the [Amim]LS-*X*.

To further investigate the pore structure of [Amim]LS-*X*, N_2_ adsorption–desorption isothermal measurements were performed. The isothermal and the corresponding pore size distribution curves were shown in Fig. 3a and b, and the plots in Fig. 3(a) can be classified as type II based on the Brunauer classification. With the increase of heat treatment temperature, the specific surface area and total pore volume of the [Amim]LS-*X* increase as well. In detail, the curves all exhibit slight downwards inflections at low potential region (*P*/*P*_0_ < 0.1), hysteresis loops (*P*/*P*_0_ in the range of 0.1–0.9), and sharp upward peaks at high pressures (>0.9 *P*/*P*_0_), demonstrating the presence of few micropores, a huge amount of mesopores and large pores in the samples.^33^

The porosity parameters of [Amim]LS-*X* carbon samples are summarized in Table 1, and the parameters for [Amim]Cl-700 and SLS-700 are also listed for comparison. It is believed that with the introduction of the N-containing small molecular IL ([Amim]Cl) to the macromolecular SLS, a new ionic liquid [Amim]LS is produced through anion exchange reaction, with NaCl as the by-product. By direct carbonization of the reaction products at different temperatures, N-doped [Amim]LS-*X* carbon materials can be obtained without any extra activation agent or template. Both the specific surface area and pore structures of the derived [Amim]LS-*X* carbon materials are between the two precursors (SLS and [Amim]Cl) (Table 1). As the carbonization temperature increased from 600 °C to 900 °C, the specific surface area of the carbon materials increased from 83 m^2^ g^−1^ to 502 m^2^ g^−1^, and the pore volume increased from 0.033 cm^3^ g^−1^ to 0.183 cm^3^ g^−1^, with the majority pore structures belonging to mesopores, which can accelerate ion diffusion at high current densities applied.^10^ Although the average pore size is much larger for [Amim]LS-600 (16.552 nm), the total pore volume is very low instead (0.033 cm^3^ g^−1^), demonstrating fewer pores available in [Amim]LS-600 than other [Amim]LS-*X* materials. A dramatic increase in both the specific surface area and micropore volume from [Amim]LS-800 to [Amim]LS-900, which is believed to be as a result of the activation of NaCl. With the temperature higher than 801 °C (the melting point of NaCl), the fast diffusion of molten NaCl into the carbon can promote the formation of more micropores and larger specific surface area, while with temperature lower than 801 °C, the activation role of NaCl is limited, which is in agreement with our results (Table 2). In fact, as previously reported, other possible inorganic salts (Na_2_SO_4_ or Na_2_CO_3_) (due to the thermal decomposition from SLS) may also exist during the carbonization and contribute to the pore development, but these are not detected by the XRD in our study. Considering the massive presence of NaCl, it is considered that NaCl plays the major role as the self-template/self-activation for [Amim]LS-*X* samples. The carbon material yields of the precursors are also evaluated and compared (Table 1). The carbon material yields of [Amim]LS-*X* are in the range of 27.4–34.6%, which is higher than those carbon materials obtained with KOH or molten salts activation.^4^

**Table 1:** Porosity parameters and carbon material yield of the samples

Entries	Sample	BET surface area^a^ (m^2^ g^−1^)	Total pore volume^b^ (cm^3^ g−1)	Micropore volume^c^ (cm^3^ g^−1^)	The percentage of micropore volume	Average pore size^d^ (nm)	Yield (wt%)
1	[Amim]Cl-700	5	0.004	0.0017	0.42	37.551	40.2
2	SLS-700	649	0.420	0.0978	0.23	3.184	29.2
3	[Amim]LS-600	83	0.033	0.0234	0.71	16.552	28.9
4	[Amim]LS-700	193	0.072	0.0585	0.81	11.122	34.6
5	[Amim]LS-800	263	0.119	0.0551	0.46	14.148	30.2
6	[Amim]LS-900	502	0.183	0.1400	0.76	11.159	27.4

a Surface area (BET) calculated using the BET method.

b Total pore volume calculated using the DFT model.

c Micropore volume calculated using the DFT model.

d Average pore size calculated using the BJH model.

**Table 2:** Surface elemental compositions of the prepared carbon samples

Entries	Sample	By XPS analysis (at%)									
		C	N	O	N-6	N-5	N-Q	N-X	O-I	O-II	O-III
1	[Amim]Cl-700	82.04	9.63	8.33	42.06	34.58	23.36	—	36	48	16
2	SLS-700	89.87	0.84	8.06	—	—	—	—	41.33	48	10.67
3	[Amim]LS-600	82.39	4.68	12.37	17.02	36.17	46.81	—	—	8.65	91.35
4	[Amim]LS-700	84.31	4.18	9.66	28.09	49.44	14.60	7.80	31.71	43.90	24.39
5	[Amim]LS-800	86.41	3.02	10.36	25.97	46.75	14.29	12.99	35.21	42.25	22.53
6	[Amim]LS-900	86.30	2.08	11.54	20.32	47.59	21.39	10.70	33.5	40.63	25.62

**Fig. 4:**
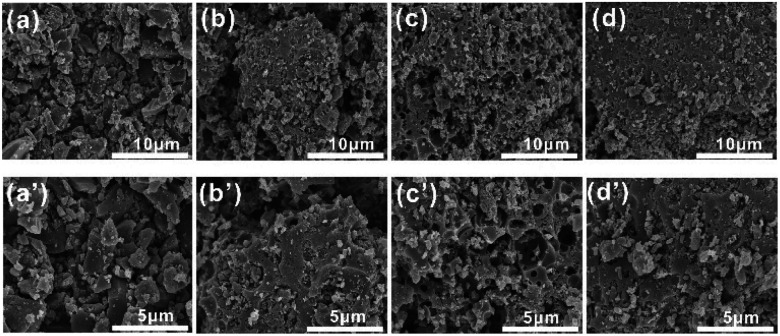
SEM images of the samples: (a) and (a′) [Amim]LS-600, (b) and (b′) [Amim]LS-700, (c) and (c′) [Amim]LS-800 and (d) and (d′) [Amim]LS-900.

The porous structural features of the as-prepared samples for [Amim]LS-*X* were confirmed by SEM. As illustrated in Fig. 4(a) and (a′), [Amim]LS-600 shows sheet-like morphology without any pore structure. When the pyrolysis temperature increases to 700 °C (Fig. 4(b) and (b′)), a large number of porous structures began to appear, and there are more pores formed with the increase of heat treatment temperature (Fig. 4(c) and (c′), Fig. 4(d) and (d′)), which demonstrates that increasing the heat treatment temperature can facilitate the pore formation, just like a typical activation process, in which the inorganic salt NaCl was considered to play a major role in the development of pore structures.

In order to explore the influence of the element content on the material properties, the surface element compositions of the samples were determined by XPS. As shown in Fig. S2,† all the three peaks C 1s, N 1s and O 1s are observed on the XPS spectra of [Amim]Cl-700, SLS-700 and [Amim]LS-*X* carbon samples. Compared with other samples, SLS-700 shows a much smaller N 1s peak, indicative of the lower N content in the sample. The quantitative analysis in Table 2 elucidates that the [Amim]LS-*X* has a high N content, with the introduction of N-rich [Amim]Cl into the SLS structure. As the carbonization temperature increases from 600 °C to 900 °C, the content of N element in [Amim]LS-*X* decreases correspondingly with a value of 4.68%, 4.18%, 3.02% and 2.08%, respectively, and the content of O element only changes slightly with a value of 12.37%, 9.66%, 10.36%, 11.54%, respectively.

**Fig. 5:**
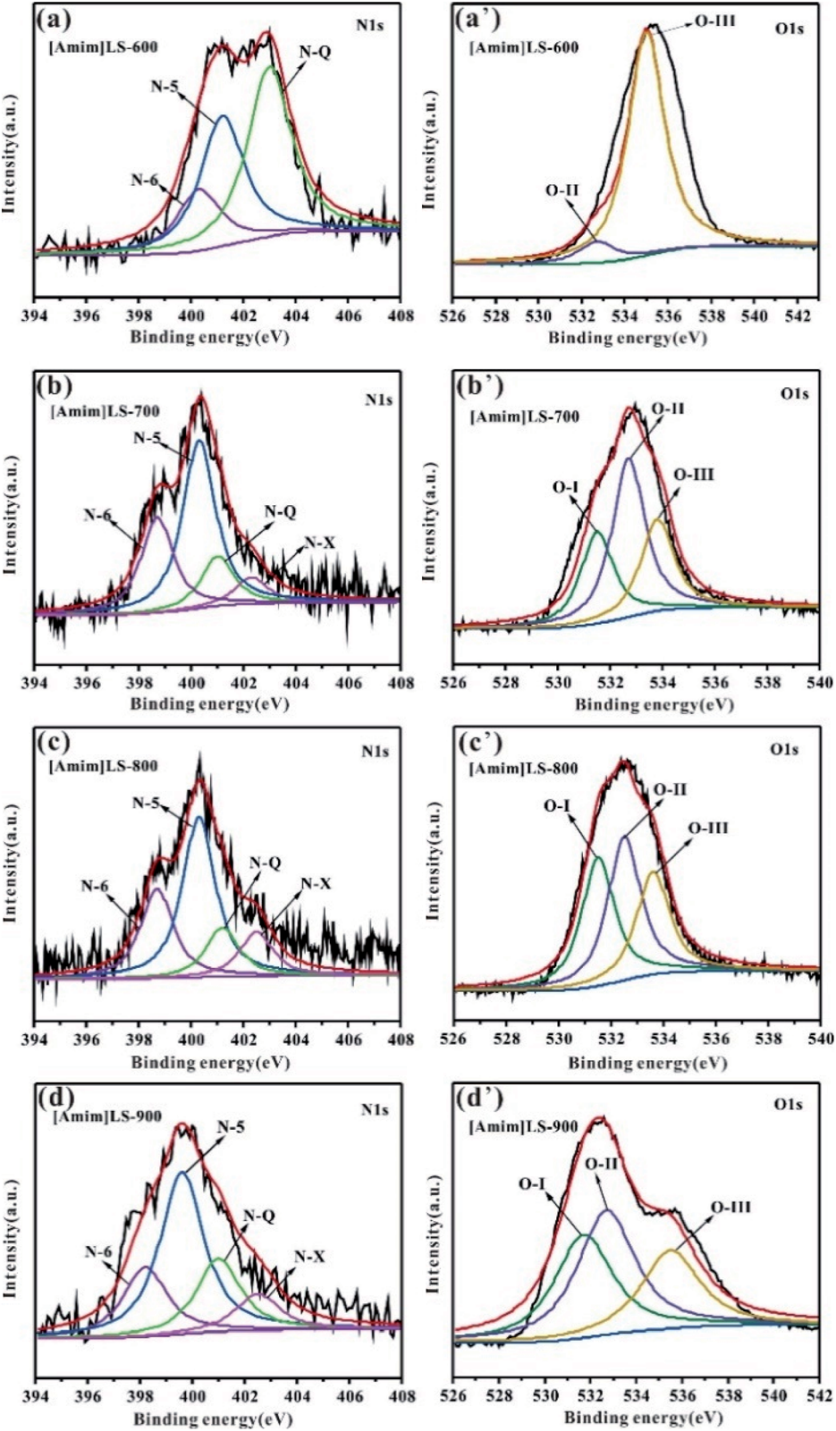
High-resolution XPS spectra of the N 1s peak of (a) [Amim]LS-600; (b) [Amim]LS-700; (c) [Amim]LS-800; (d) [Amim]LS-900 and O 1s peak of (a′) [Amim]LS-600; (b′) [Amim]LS-700; (c′) [Amim]LS-800; (d′) [Amim]LS-900.

To further understand the chemical states of N and O elements in the sample, the high resolution XPS spectra of N 1s and O 1s were shown in Fig. 5. The high resolution N 1s spectra of the [Amim]LS-*X* samples can be fitted into four individual peaks representing pyridine nitrogen (N-6, 398.2–398.7 eV), pyrrolic/pyridine nitrogen (N-5, 399.2–400.3 eV), quaternary nitrogen (N-Q, 401.2–401.7 eV) and oxidized pyridine nitrogen (N-X, 402.3–403.1 eV), respectively.^9,52–54^ It can be seen from Table 2 that the total content of N-6 and N-5 for [Amim]LS-*X* decrease from 77.53% to 67.91%, while those of N-Q and N-X increase from 22.4% to 32.09%, as the temperature deceases from 700 °C to 900 °C. However, there is no N-6 detected on [Amim]LS-600, although which has a value of up to 82.89% of N-Q and N-X. The content of N-6, N-5 and N-Q are 42.06%, 34.58% and 23.36%, respectively, for [Amim]Cl-700. It is well accepted that the N-containing functional groups such as N-5 and N-6 are considered to be favorable for electrochemical performance by contributing to the pseudo-capacitance,^55^ while N-Q and N-X play an important role in enhancing the electronic conductivity of the carbon structures.^56^ Similarly, the high resolution O 1s spectra can be divided into three peaks, which corresponds to the C–O groups (O-I, 531.3–531.5 eV), C–OH/C–O–C groups (O-II, 532.3–532.7 eV) and –COOH groups (O-III, 533.5–534.0 eV). It was found that C–OH/C–O–C groups and –COOH groups on the carbon skeleton may be more beneficial in alkaline electrolyte.^32,45^ The content of O-II and O-III in the carbon samples follows the order of [Amim]LS-600 > [Amim]LS-700 > [Amim]LS-900 > [Amim]LS-800. The presence of abundant N-oxide groups in carbon materials can improve the surface wettability of the materials, thus promoting the interaction between the electrode material and the electrolyte.

**Fig. 6:**
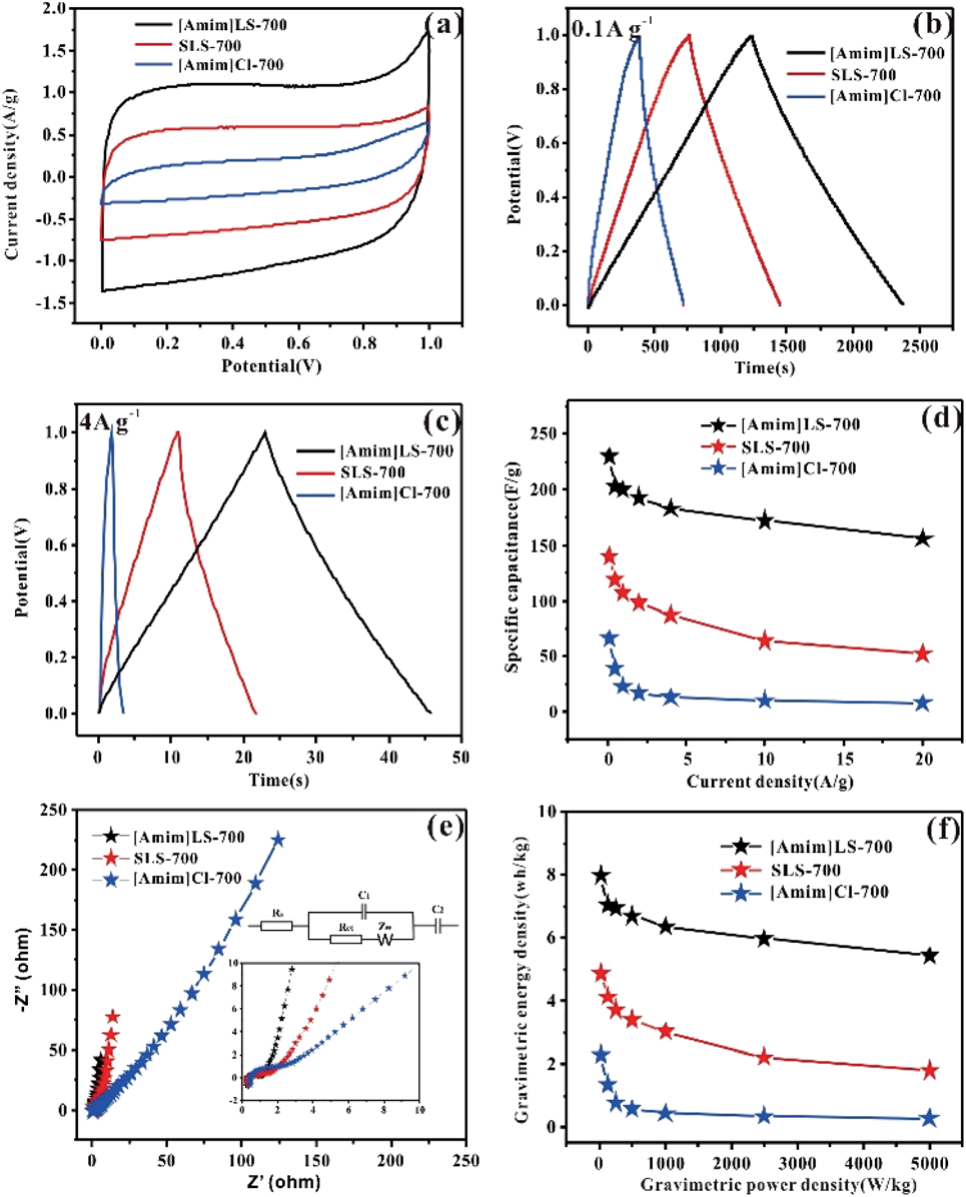
Electrochemical performances of electrode in 6 mol L^−1^ KOH: (a) cyclic voltammetry of SLS-700, [Amim]Cl-700 and [Amim]LS-700 at 10 mVs^−1^; (b) GCD curves of the SLS-700, [Amim]Cl-700 and [Amim]LS-700 at 0.1 A g^−1^; (c) GCD curves of the SLS-700, [Amim]Cl-700 and [Amim]LS-700 at 4 A g^−1^; (d) the specific capacitances calculated from the discharge curves at different current densities; (e) typical Nyquist plot and the equivalent circuits of the SLS-700, [Amim]Cl-700 and [Amim]LS-700 samples; (f) the energy and power density of SLS-700, [Amim]Cl-700 and [Amim]LS-700 samples.

The electrochemical properties of the as prepared carbon materials as electrodes in supercapacitors were measured in a two-electrode system with 6 M KOH aqueous electrolyte, with the voltage window of 0–1 V. For a comparative purpose, the electrochemical performances of SLS-700, [Amim]Cl-700 and [Amim]LS-700 samples are evaluated firstly. Fig. 6(a) shows the cyclic voltammetry of SLS-700, [Amim]Cl-700 and [Amim]LS-700 at a scan rate of 10 mV s^−1^. [Amim]LS-700 exhibits much higher current density than that of SLS-700 and [Amim]Cl-700, indicating the positive effect of the introduction of IL to SLS on the specific capacitance. The CV curves at different scanning rates for the samples in Fig. S4† reveal that [Amim]LS-700 can maintain good rectangular shape even at a scan rate of 200 mV s^−1^ compared with [Amim]Cl-700 and SLS-700, suggesting superior double-layer electron transfer behaviour of [Amim]LS-700. Similarly, the GCD curves at 0.1 A g^−1^ and 4 A g^−1^ of [Amim]LS-700 exhibit symmetrical triangular shapes as shown in Fig. 6(b) and (c), implying its perfect electrochemical rever sibility and coulomb efficiency. The specific capacity of [Amim]LS-700 is 230 F g^−1^ at 0.1 A g^−1^, which is much higher those that of [Amim]Cl-700 (66 F g^−1^) and SLS-700 (140 F g^−1^). Increasing the current density to 4 A g^−1^ results in a decrease in their specific capacity to values of 183 F g^−1^, 13 F g^−1^ and 87 F g^−1^, in the cases of [Amim]LS-700, [Amim]Cl-700, respectively.

It is worthy to mentioning that the specific surface area of SLS-700 (649 m^2^ g^−1^) is much higher than that of [Amim]LS-700 (193 m^2^ g^−1^), but the N content of SLS-700 is only 0.84%, while that of [Amim]LS-700 is 4.071%. The observed superior electrochemical properties of [Amim]LS-700 can be explained by its higher content of nitrogen and oxygen groups, which makes the electrode material change its affinity to electrolyte, thus increasing the ion transfer rate and improving the internal redox reaction.^57,58^ Although the N content of [Amim]Cl-700 is as high as 9.627%, it has extremely low specific surface area of 5 m^2^ g^−1^, therefore, a low mass specific capacity of 13 F g^−1^ is achieved as it is unable to store the charge transferred by the electrolyte. The capacitance retention of the samples at the current density of 0.1–20 A g^−1^ were studied and the results were shown in Fig. 6(d), it is found that [Amim]LS-700 has a much higher capacitance retention of 65% than those of SLS-700 (37.14%) and [Amim]Cl-700 (12%) at 20 A g^−1^.

Fig. 6(e) exhibits the Nyquist plots of [Amim]LS-700, SLS-700 and [Amim]Cl-700 samples, and the inset shows the enlarged detail for high frequency region. It is obvious that all the samples present ideal capacitive behaviour at high frequency region with small semicircles. The intercept of the curves represents the equivalent resistance (*R*_ct_) corresponding to the charge transfer process of the electrode/electrolyte interface. The line of the [Amim]LS-700 in the low-frequency is more nearly parallel to the *Y*-axis than those of SLS-700 and [Amim]Cl-700, which indicates that the [Amim]LS-700 sample presents the dominance of double-layer charge storage with faster charge transfer characteristics. As shown in Fig. 6(f), the energy density reaches 7.99 W h kg^−1^ at the power density of 25 W kg^−1^ for [Amim]LS-700, which is much higher than those of SLS-700 (4.86 W h kg^−1^ at 25.43 W kg^−1^) and [Amim]Cl-700 (2.29 W h kg^−1^ at 24.85 W kg^−1^).

**Fig. 7:**
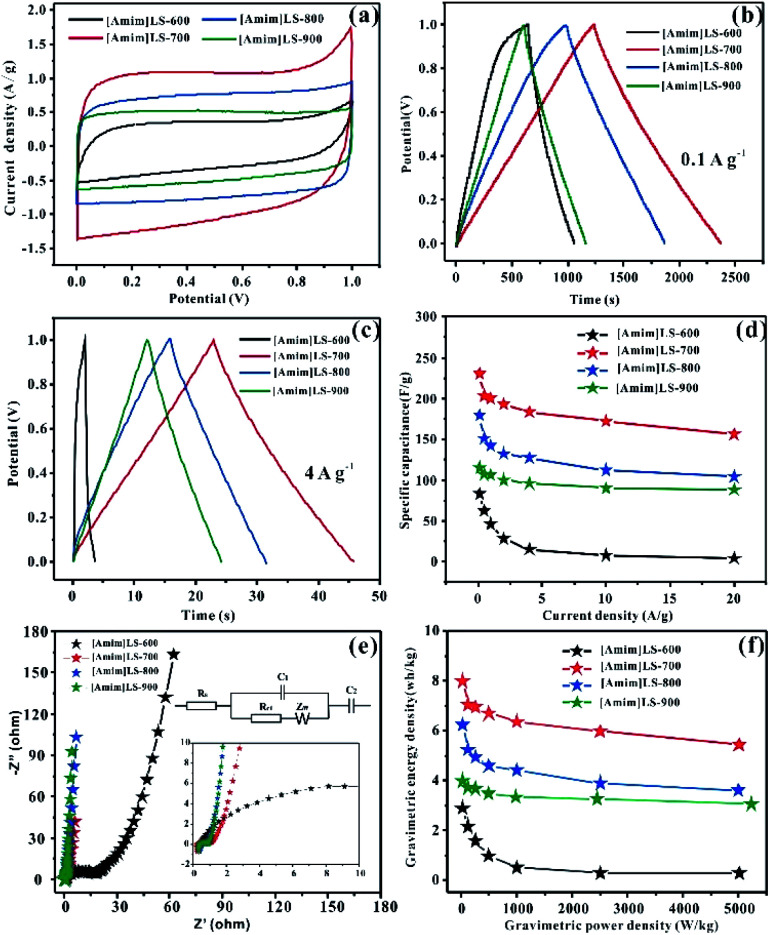
Electrochemical performances of [Amim]LS-*X* carbon materials in 6 mol L^−1^ KOH: (a) CVs at 10 mV s^−1^; (b) GCD curves at 0.1 A g^−1^; (c) GCD curves at 4 A g^−1^; (d) the specific capacitances calculated from the discharge curves at different current densities; (e) typical Nyquist plot and the equivalent circuits of the [Amim]LS-*X* samples; (f) the energy and power density of [Amim]LS-*X* samples.

The influence of the pyrolysis temperature on the electrochemical performance of [Amim]LS-*X* samples is shown in Fig. 7. All the CV curves of [Amim]LS-*X* samples present a rectangular shape at the scanning rate of 10 mV s^−1^ (Fig. 7(a)), implying their typical double-layer capacitance characteristics. [Amim]LS-700 has the best electrochemical capacitance, which is evidenced by its largest area of CV loop and the highest current density. The GCD curves of the as-prepared carbon materials are presented in Fig. 7(b) (at 0.1 A g^−1^) and Fig. 7(c) (at 4 A g^−1^). It is found that isosceles triangle shapes are observed in the cases of [Amim]LS-700, [Amim]LS-800, [Amim]LS-900, while a little deformation of the isosceles triangle shape is observed in the case of [Amim]LS-600. The deformation can be explained by its low specific surface area (5 m^2^ g^−1^), and it is difficult to transfer the ions from electrolyte to the active material. The symmetrical triangles of the samples reveal that the samples have good coulomb efficiency and electric double layer charge transfer as well as good electrochemical reversibility. Based on the GCD curves, the specific capacitance for [Amim]LS-700 are calculated as 230 F g^−1^ at 0.1 A g^−1^, which is much better than that of [Amim]LS-600 (83 F g^−1^), [Amim]LS-800 (179 F g^−1^) and [Amim]LS-900 (115 F g^−1^). Similar tendency is observed with a current density at 4 A g^−1^, and the specific capacitance is 183 F g^−1^, 15 F g^−1^, 127 F g^−1^ and 96 F g^−1^ in the cases of [Amim]LS-700, [Amim]LS-600, [Amim]LS-800 and [Amim]LS-900, respectively. These comparative results indicate that their specific capacitance is determined by the synergistic effect of both the specific surface area and the doped heteroatom.^59,60^ Although the specific surface area and pore volume of [Amim]LS-700 (193 m^2^ g^−1^, 0.072 cm^3^ g^−1^) are not the highest among the [Amim]LS-*X* samples, actually much lower than those of [Amim]LS-900 (502 m^2^ g^−1^, 0.183 cm^3^ g^−1^), the N content of [Amim]LS-700 (4.18 at%) and both of the contents of N-6 and N-5 on the surface of [Amim]LS-700 are highest (Table 2).

Fig. 7(d) illustrates their capacitance retention at current density from 0.1–20 A g^−1^. The capacitance retention is ∼4.8%, ∼68%, ∼63% and ∼75% for [Amim]LS-600, [Amim]LS-700, [Amim]LS-800 and [Amim]LS-900, respectively. Interestingly, although [Amim]LS-700 exhibits the largest value of specific capacitance, [Amim]LS-900 sample shows the highest capacitance retention of 75%. It is well accepted that the higher content of nitrogen not only increases the ion transfer rate, but also improves the internal redox reaction to improve the pseudo-capacitance.^61^ The high capacitance retention for [Amim]LS-900 may be attributed to its favourable pore structures, thus endowing efficient charge–discharge process, and the high proportion of N-Q and N-X content may increase its hydrophobicity, thus accelerating electron transfer of carbon materials. It can also be found in the CVs study at 20 mV s^−1^ -200 mV s^−1^ for [Amim]LS-*X* (Fig. S4†), [Amim]LS-900 exhibits the greatest rectangular shape, further indicating its good capacity retention performance. EIS was performed to investigate the frequency response characteristics of [Amim]LS-*X*, and the results were shown in Fig. 7(e). All the samples show ideal capacitive behaviour at the high frequency region with small semicircles. It is found that a much smaller semicircle diameters is observed in the cases of [Amim]LS-700, [Amim]LS-800 and [Amim]LS-900, exhibiting lower contact resistance and excellent conductivity. The line of the [Amim]LS-900 at the low-frequency is more nearly parallel to the *Y*-axis than other samples, demonstrating its good conductivity and best characteristic double-layer charge storage, which may be due to its high content of N-Q (21.39 at%) and N-X (10.70 at%). The equivalent circuits of the carbon samples are listed in the inset of Fig. 6(e) and 7(e), and the detailed (*R*_s_, *R*_ct_) values are calculated for all the carbon samples (Table S1†), which is related to the graphitization degree and surface functionalities. Energy density and power density are important parameters for industrial application of supercapacitors in energy storage. Fig. 7(f) shows the Ragone plots of energy density and power density for [Amim]LS-*X* samples. As expected, [Amim]LS-700 has the highest energy density of 7.99 W h kg^−1^ when the power density is 25 W kg^−1^; at high power density of 5003.8 W kg^−1^, the energy density is 3.9 W h kg^−1^. The comparison of the electrochemical performance of [Amim]LS-700 with those in some previously publications (Table S2†) demonstrates the as-prepared carbon materials has potential application in supercapacitors.

**Fig. 8:**
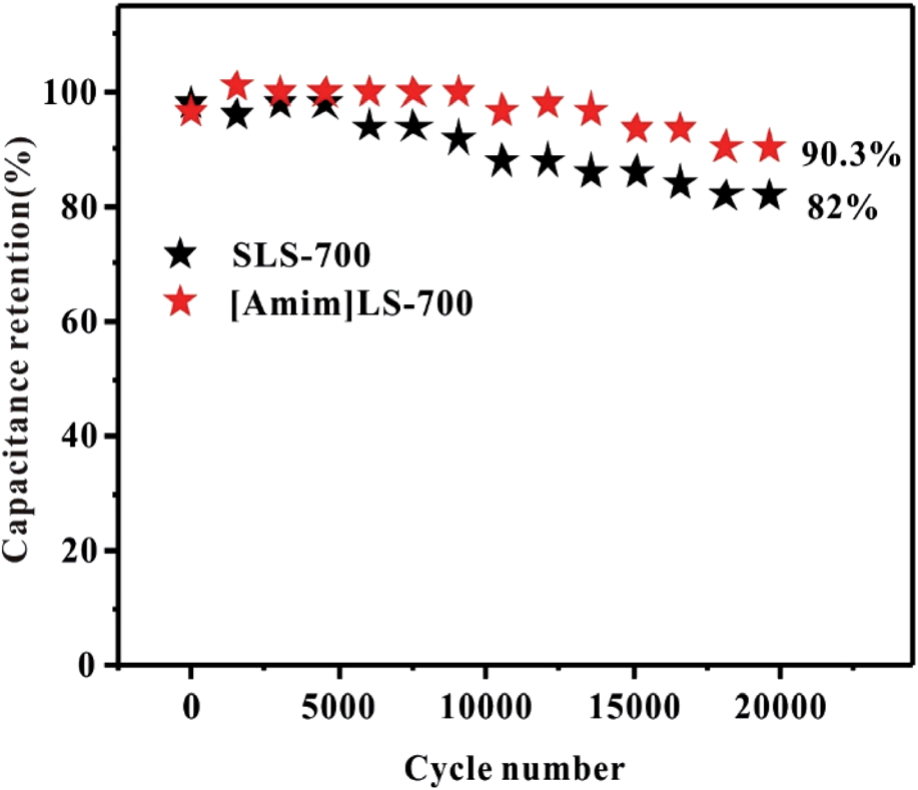
Cycling stability of [Amim]LS-700 and SLS-700 at a current density of 2 A g^−1^ for 20 000 cycles in 6 M KOH electrolyte.

Cycling stability is another important indicator in practical applications for supercapacitors. As shown in Fig. 8, the cycling stability of [Amim]LS-700 and SLS-700 under the current density at 2 A g^−1^ was investigated. It can be observed that at the beginning stage, the capacitance retention is very high and there is no obvious difference between the two samples. With the increase of the cycling, [Amim]LS-700 still presents outstanding capacitance retention up to 90.3% after 20 000 cycling, much higher than that of 82% for SLS-700. Furthermore, the approximately 100% coulombic efficiency indicates the excellent charge–discharge reversibility of [Amim]LS-700 (Fig. S5†).

## Conclusions

Herein, a high N-doped porous carbon was easily prepared by using lignosulfonate based ILs as precursor which was prepared by a simple ion exchange reaction between sodium lignosulfonate and 1-allyl-3-methyl imidazolium chloride, and the *in situ* produced NaCl was used as a self-template and self-activation agent. It was found that the pyrolytic temperature had important effect on the physical, chemical structures as well as electrochemical properties of the carbon materials, and [Amim]LS-700 exhibiting the highest capacity of 230 F g^−1^ at the current density of 0.1 A g^−1^, a satisfactory energy density of 7.99 W h kg^−1^ at the power density of 25 W kg^−1^, and satisfactory cycling stability of 90.3% capacitance retention after 20 000 cycles at the current density of 2 A g^−1^. Furthermore, the yield of [Amim]LS-700 carbon material is up to 34.6%, which is much superior to that of most carbon materials obtained using traditional activation agents. In words, the findings in this study provided a facile method to use sodium lignosulfonate, a by-product from traditional pulp making industry, as a raw material for energy materials preparation.

## Conflicts of interest

There are no conflicts to declare.

## Supplementary Material

RA-010-D0RA06821G-s001
