# Supplementary material for: Self-template/activation nitrogen-doped porous carbon materials derived from lignosulfonate-based ionic liquids for high performance supercapacitors
Source: RSC Adv. 2020 Oct 5;10(60):36504–13. doi: 10.1039/d0ra06821g (PMC9057050; doi:10.1039/d0ra06821g)
Supplement: RA-010-D0RA06821G-s001 [file RA-010-D0RA06821G-s001.pdf]

Electronic Supplementary Material (ESI) for RSC Advances.
This journal is © The Royal Society of Chemistry 2020

Supporting information for
Self-template/activation nitrogen-doped porous carbon materials derived from lignosulfonate for high performance supercapacitors

Qinqin Xu[#], Xia Wang,[#] Jian Cheng, Lin Zhang, Feng He, Haibo Xie*

Department of New Energy Science & Engineering, College of Materials & Metallurgy, Guizhou University, Huaxi District, Guiyang 550025, P.R.China

***Corresponding Authors:**

Haibo Xie, E-mail: hbxie@gzu.edu.cn

1. Electrochemical measurements

The gravimetric capacitance (C_g, F g^{-1}) of a single electrode is calculated from the discharge curves according to the following equation (1) [1-4]:

$$C_{g=}\frac{2I\,\Delta\,t}{m\,\Delta\,V} \quad (1)$$

where I (A) is the constant discharge current, Δt (s) the discharge time, m (g) the mass of the active material in a single electrode, and ΔV (V) the voltage change in discharge.

The gravimetric energy density (W_g, Wh kg^{-1}) and power density (P_g, W kg^{-1}) of the two-electrode symmetric supercapacitors are also calculated according to equation (2 & 3):

$$W_g = \frac{C_g \Delta V^2}{8x3.6} \quad (2)$$

$$P_g = \frac{3600xWg}{\Delta t} \quad (3)$$

where C_g (F g^{-1}) represents the gravimetric specific capacitance of a single electrode obtained from equation (1), ΔV (V) the voltage change in discharge, and Δt (s) the discharge time.

2. Figures

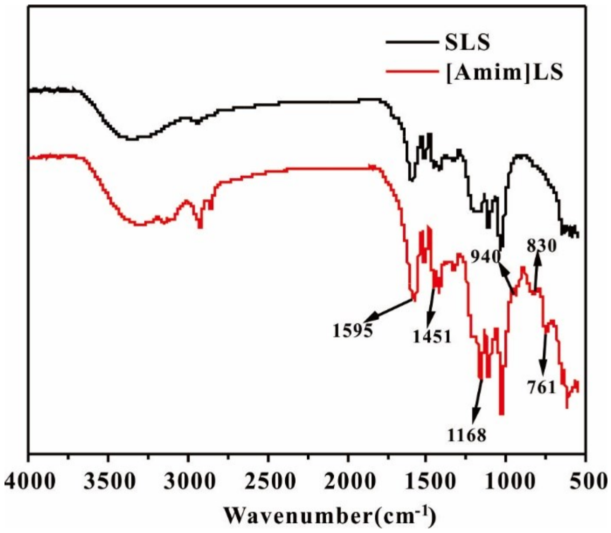

Figure S1. FTIR spectra of SLS and [Amim]LS precursor

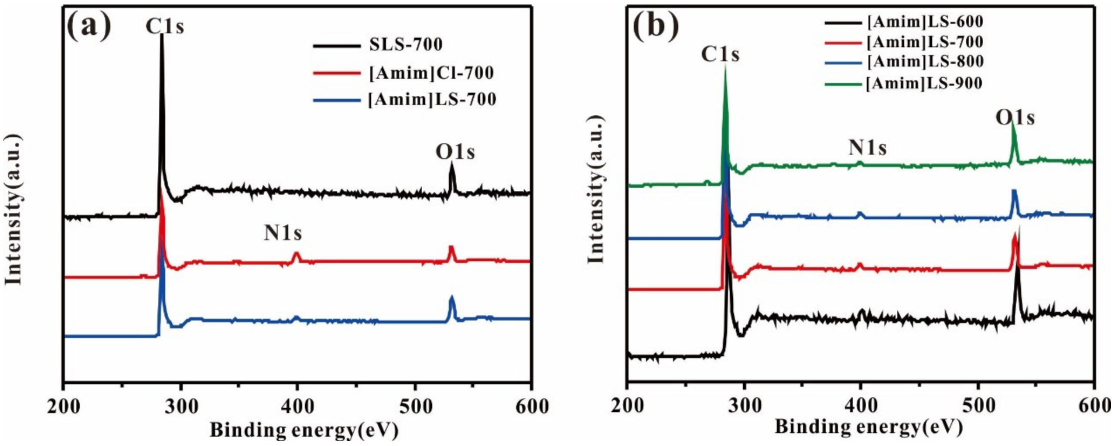

Figure S2. Wide-scan XPS spectra of (a) SLS-700, [Amim]Cl-700 and [Amim]LS-700 carbon samples, (b) carbon samples at different preparation temperatures (600 ℃ - 900 ℃) ([Amim]LS-600, [Amim]LS-700, [Amim]LS-800 and [Amim]LS-900)

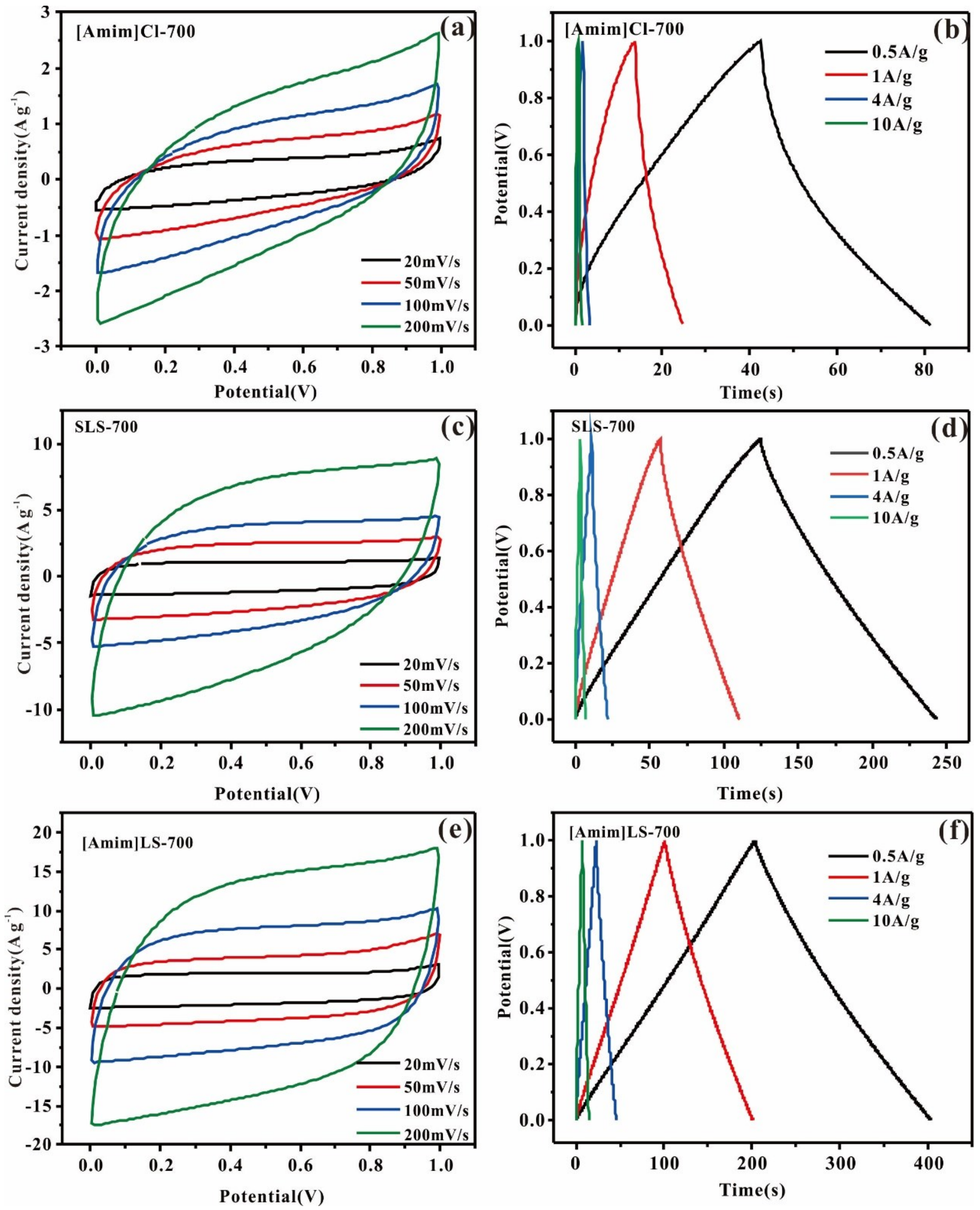

Figure S3. (a) (c) (e) CV curves at 20 mV s^{-1} - 200 mV s^{-1} and (b) (d) (f) GCD curves at 0.5 A g^{-1} ~ 10 A g^{-1} of SLS-700, [Amim]Cl-700 and [Amim]LS-700 carbon samples in 6 mol L^{-1} KOH

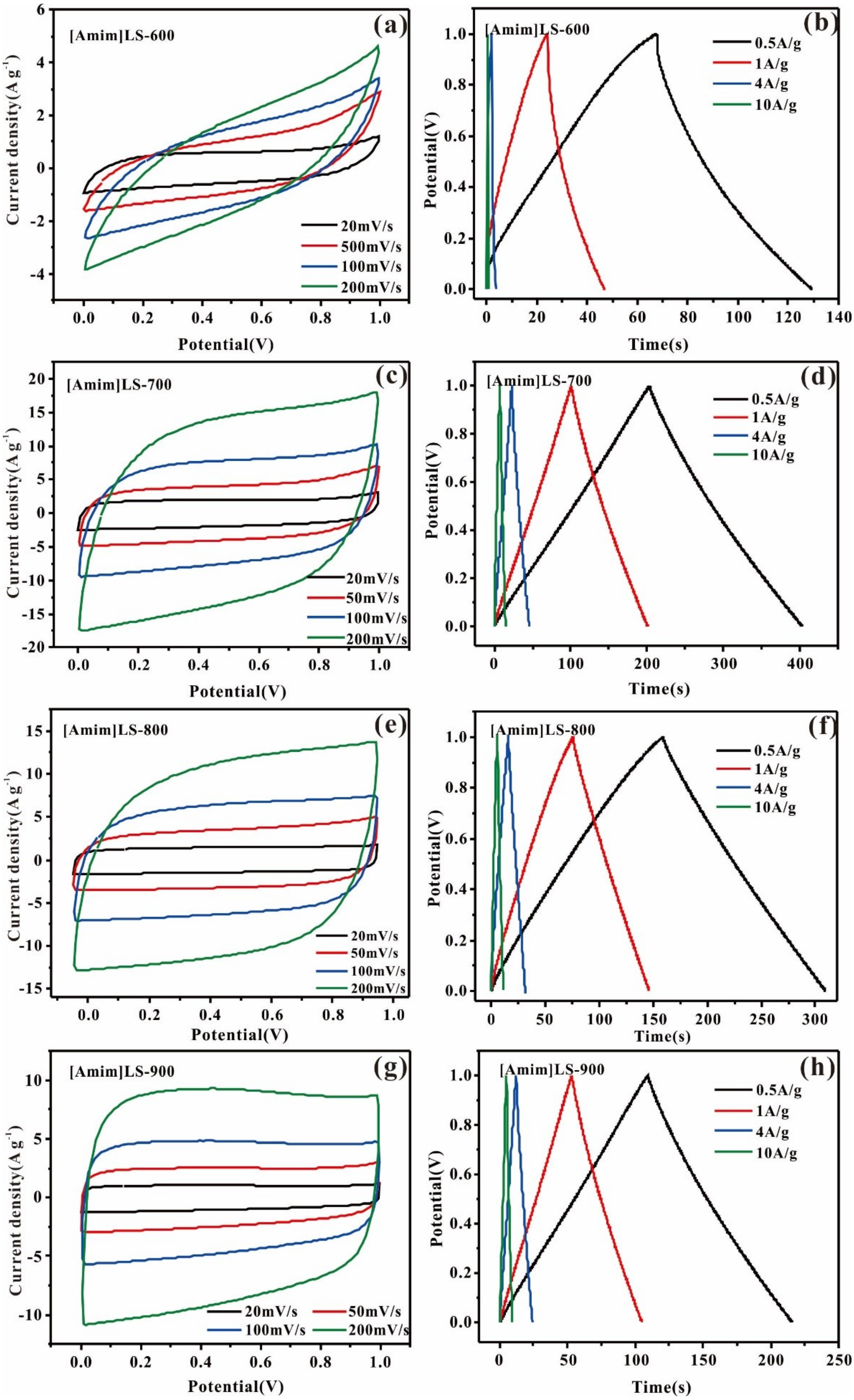

Figure S4. (a) (c) (e) (g) CV curves at 20 mV s^{-1} ~ 200 mV s^{-1} and (b) (d) (f) (h) GCD curves at 0.5 A g^{-1} ~ 10 A g^{-1} of [Amim]LS-X carbon samples in 6 mol L^{-1} KOH

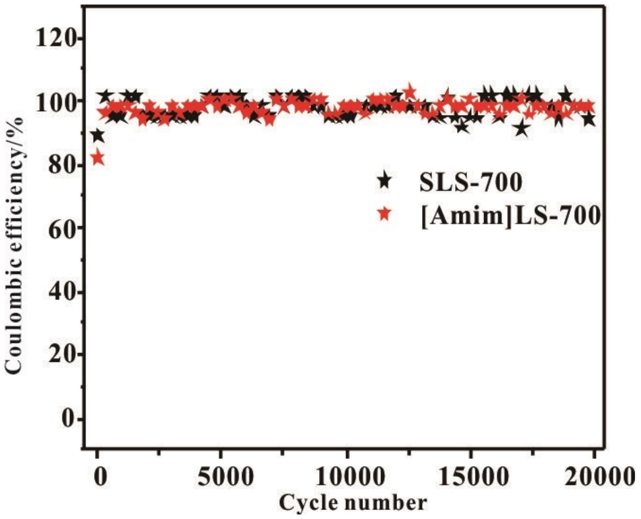

Figure S5. Coulombic efficiency of SLS-700 and [Amim]LS-700 carbon samples

Table S1 The calculated parameters of the equivalent circuit for the obtained carbon samples

	[Amim]LS-600	[Amim]LS-700	[Amim]LS-800	[Amim]LS-900	SLS-700	[Amim]Cl-700
Rs (Ω)	0.489	0.293	0.473	0.373	0.334	0.472
Rct(Ω)	15.661	0.713	0.25	0.499	1.171	1.234

Table S2 Comparison of the supercapacitance performance in this work and other literatures

**Electrode**	**Electrolyte**	**Frequency range**	**Potential window (V)**	**Current density (A g^{-1})**	**Specific capacitance (F g^{-1})**	**Energy density (Wh kg^{-1})**	**Power density (W kg^{-1})**	**Refs**
Lignosulfonate derived hierarchical porous carbons	7 M KOH	0.01Hz~100k Hz	0 – 1	0.05	247	3.4	6.18	5
Molten salt synthesis of nitrogen-doped carbon	1 M KOH	0.01Hz~100k Hz	-1 – 0	0.5	252	8.75	125	6
Nitrogen-doped ordered mesoporous carbons	6 M KOH	0.01Hz~100k Hz	-0.9 - 0.1	0.5	230	5.11	100	7
Sustainable nitrogen-		0.01Hz~100k						

containing hierarchical porous carbon		Hz						
Biomass derived interconnected hierarchical micro-meso-macro- porous carbon	1 M H_2SO_4	0.01Hz~100k Hz	-0.1 - 0.9	0.2	550	19.09	50	9
Fishbone-derived N-doped hierarchical porous carbon	1 M H_2SO_4	0.01Hz~100k Hz	0 – 1.0	0.1	476	21.2	39	10
Nitrogen-Doped Porous Carbon	6 M KOH	0.01Hz~100k Hz	0 - 1.0	1	202	7.01	250	11
**[Amim]LS-700**	**6 M KOH**	**0.01Hz~100k Hz**	**0 - 1.0**	**0.1**	**230**	**7.99**	**25**	**This work**

Ref:

1. J.-w. Lang, X.-b. Yan, W.-w. Liu, R.-t. Wang and Q.-j. Xue, *J. Power Sources*, 2012, **204**, 220-229.
2. L. Sun, C. G. Tian, M. T. Li, X. Y. Meng, L. Wang, R. H. Wang, J. Yin and H. G. Fu, *J. Mater Chem. A*, 2013, **1**, 6462-6470.
3. G. Wang, J. Huang, S. Chen, Y. Gao and D. Cao, *J. Power Sources*, 2011, **196**, 5756-5760.
4. N. Jha, P. Ramesh, E. Bekyarova, M. E. Itkis and R. C. Haddon, *ADV ENERGY MATER*, 2012, **2**, 438-444.
5. J. Pang, W. Zhang, J. Zhang, G. Cao, M. Han and Y. Yang, *Green Chemistry*, 2017, **19**, 3916-3926.
6. X. Deng, B. Zhao, L. Zhu and Z. Shao, *Carbon*, 2015, 48-58.
7. Q. Shi, R. Zhang, Y. Lv, Y. Deng and D. Zhao, *Carbon*, 2015, **84**, 335-346.
8. J. Pang, W. Zhang, H. Zhang, J. Zhang, H. Zhang, G. Cao, M. Han and Y. Yang, *Carbon*, 2018, **132**, 280-293.
9. S. Yang, S. Wang, X. Liu and L. Li, *Carbon*, 2019, **147**, 540-549.
10. J. M. A, S. I. W. B, Q. L. A, P. Z. A, J. Z. A, Y. Z. A, J. S. B and S. Z. A, *Journal of Alloys Compounds*, 2020, **832**.
11. L. F. Chen, X. D. Zhang, H. W. Liang, M. Kong, Q. F. Guan, P. Chen, Z. Y. Wu and S. H. Yu, *Acs Nano*, 2012, **6**, 7092-7102.
